# Budget constraint and vaccine dosing: a mathematical modelling exercise

**DOI:** 10.1186/1478-7547-12-3

**Published:** 2014-01-22

**Authors:** Baudouin A Standaert, Desmond Curran, Maarten J Postma

**Affiliations:** 1 Health Economics Department, GlaxoSmithKline Vaccines, Avenue Fleming 20, 1300 Wavre, Belgium; 2 Unit of PharmacoEpidemiology & PharmacoEconomics (PE2), Department of Pharmacy, University of Groningen, Groningen, The Netherlands

**Keywords:** Rotavirus, Vaccination, Economic evaluation, Budget optimisation modelling

## Abstract

**Background:**

Increasing the number of vaccine doses may potentially improve overall efficacy. Decision-makers need information about choosing the most efficient dose schedule to maximise the total health gain of a population when operating under a constrained budget. The objective of this study is to identify the most efficient vaccine dosing schedule within a fixed vaccination budget from a healthcare payer perspective.

**Methods:**

An optimisation model is developed in which maximizing the disease reduction is the functional objective and the constraint is the vaccination budget. The model allows variation in vaccination dosing numbers, in cost difference per dose, in vaccine coverage rate, and in vaccine efficacy. We apply the model using the monovalent rotavirus vaccine as an example.

**Results:**

With a fixed budget, a 2-dose schedule for vaccination against rotavirus infection with the monovalent vaccine results in a larger reduction in disease episodes than a 3-dose scheme with the same vaccine under most circumstances. A 3-dose schedule would only be better under certain conditions: a cost reduction of >26% per dose, combined with vaccine efficacy improvement of ≥5% and a target coverage rate of 75%. Substantial interaction is observed between cost reduction per dose, vaccine coverage rate, and increased vaccine efficacy. Sensitivity analysis shows that the conditions required for a 3-dose strategy to be better than a 2-dose strategy may seldom occur when the budget is fixed. The model does not consider vaccine herd effect, precise timing for additional doses, or the effect of natural immunity development.

**Conclusions:**

Under budget constraint, optimisation modelling is a helpful tool for a decision-maker selecting the most efficient vaccination dosing schedule. The low dosing scheme could be the optimal option to consider under the many scenarios tested. The model can be applied under many different circumstances of changing dosing schemes with single or multiple vaccines.

## Background

The initial dosing schedule of a new vaccine is based on the results obtained in randomised clinical trials which evaluate the efficacy at the individual level. When real-world data on effectiveness become available questions may be raised over whether the initial dosing schedule is the most appropriate one to achieve the maximum benefit at the population level from limited available healthcare resources. This is an interesting economic question in which the number, timing and efficacies of vaccine doses should be assessed in detail. In the analysis presented here we evaluate the impact of a change in number of vaccine doses and the economic value of such a change under the constraint of a fixed vaccine budget, a situation most likely to occur in low-income countries. We have used the monovalent rotavirus vaccine (*Rotarix*®^a^) as a concrete example as it has recently been suggested that the number of doses for this vaccine should be increased in low-income countries [1].

Rotavirus infection results in a high burden of acute gastroenteritis disease in children, especially in low-income countries, with approximately 450,000 deaths that could be prevented each year by vaccination [2]. There are currently two vaccines available against rotavirus [3], but all analyses here performed are presented with the 2-dose attenuated single human rotavirus strain monovalent rotavirus vaccine (*Rotarix*®) [4,5].

In 2009 the World Health Organization recommended the inclusion of rotavirus vaccination in routine immunization programs worldwide [6]. However, trials and observational studies conducted in low-income countries have reported lower vaccine efficacy than in high-income countries [7-9]. Many hypotheses have been formulated to explain this, but no definitive conclusions have been drawn [10,11]. Nevertheless, the morbidity and mortality impact expected in low-income countries greatly surpasses that in high-income countries, despite the lower inferred vaccine efficacy [12].

To improve the results of vaccination it has been recently suggested that adding one dose to the existing vaccine dosing schedule could improve overall vaccination efficacy [1]. However this is by no means certain as 3- dose vaccine efficacy studies with other rotavirus vaccine products tested in low-resource environments have also reported lower efficacy estimates compared with wealthier settings [13,14]. In low-income countries where healthcare budgets are tight, a 2-dose schedule could be a more efficient option than a 3-dose schedule as fewer administrations may reduce the overall vaccination cost [15]. Administration may be particularly expensive in those countries, as the costs of the logistics required to maintain a cold chain may be high [16,17]. A 2-dose schedule may also achieve improved compliance and completion of the total dosing at an earlier time point as it obviously requires fewer doses to obtain full vaccination compared with a 3-dose schedule [18].

Given the considerations above, administering an additional dose could improve the rotavirus vaccine efficacy, but it raises an economic question of whether this would provide acceptable added value. Traditional health economic analysis would calculate the incremental cost-effectiveness ratio (ICER) to explore whether the additional dose is cost-effective compared with the current 2-dose schedule. If the analysis indicates that the extra budget needed for reaching the extra benefit is acceptable under the local constraints, it then requires that extra budget is found to secure the implementation of this new intervention. However, in low-income countries there may be no extra budget available to administer the additional vaccine dose even if it would be cost-effective. In such environments, the addition of an extra vaccine dose may be possible only at the expense of cuts elsewhere in the fixed budget. Conversely, it may be possible to improve the clinical results by increasing the vaccine coverage rate without adding an extra dose. Therefore, it may be more appropriate to consider a different economic approach and to compare the clinical outcomes obtained with a 2-dose schedule with a higher vaccine coverage rate versus the clinical outcomes obtained with a 3-dose schedule at a higher vaccine efficacy but a lower coverage rate. In other words, given a fixed budget, when would it be efficient to move to a 3-dose strategy? The solution to this question is no longer driven by a cost-effectiveness threshold but by the fixed budget: what is the best way to spend money under a fixed budget in order to obtain a maximum health benefit? This type of question can best be analysed at the population level (accumulated benefit and cost), in contrast to cost-effectiveness analysis that can be assessed at the individual level. It also seems to be a realistic way for local decision-makers to evaluate the benefit of vaccination strategies [19].

In this paper we evaluate the potential cost and health effect of adding a third dose to the existing 2-dose schedule of the monovalent rotavirus vaccine in low-income countries, using a hypothetical model to explore this. The model uses optimisation theory, in which a wide range of scenarios are explored to find the optimum solution under budget constraint. In sensitivity analysis, we investigate the influence of several variables on the results, including vaccine efficacy, coverage rate, and price per dose.

## Methods

The economic question raised in the introduction, “what is the best way to spend a fixed budget to obtain the maximum health benefit from vaccination?” can best be explored using optimisation or mathematical programming models [20]. The exercise is to reach specific (functional) objectives or goals under certain constraints. In this setting, the objective function is to maximise health benefits. The model has been programmed to evaluate just one particular disease with one intervention type, but different diseases with different interventions assessing a same outcome could be considered as well.

In the particular case of rotavirus disease, the outcome measure, used to assess the benefit, is the total number of diarrhoea events in the population of children aged <5 years, and the objective is to minimise the number of such events. As a direct consequence of this, mortality and hospitalisation rates due to rotavirus disease would also be reduced. We conducted a cross-sectional analysis with an annual budget, estimating events per year in the at-risk population at steady-state. The latter typically reflects the situation when disease spread and vaccine efficacy have reached their equilibrium across the entire at-risk population. The model constraints are:

• Annual vaccination budget is fixed;

• Vaccine efficacy for a 3-dose strategy ≥ than that for a 2-dose strategy;

• Vaccine efficacy for a 3-dose strategy < 150% of that for a 2-dose strategy;

• Cost per dose for a 3-dose strategy < than that for a 2-dose strategy;

The model assumes a fixed cost per dose for the administration and for the logistics to maintain the cold chain. The coverage rate allows a variation between 0% and 100%. No discounting is applied as it concerns a budget analysis.

The model construct is developed in Microsoft *Excel*, using additional Solver tools (Frontline Systems, Inc.) from software specifically designed to be integrated as an add-in into Microsoft *Excel*. The results of the optimisation model indicate which strategy (i.e. a 2- or 3-dose strategy) would produce maximum health benefits under a budget constraint. The analysis is conducted from the perspective of the healthcare payer system. A copy of the model is available as a Microsoft *Excel* spread sheet (see Additional file 1).

As the current exercise is hypothetical we do not apply it to a specific country. The whole analysis is focussed on the relationships between the critical variables and their relative values.

Sensitivity analysis is conducted by varying three key parameters that affect the results, vaccine efficacy, price, and coverage. The relationships between the variables are as follows: the number of overall diarrhoea events avoided by a 2-dose schedule (y) is a function of the vaccine coverage rate (a), the vaccine efficacy (x) obtained, and the disease population incidence rate (i):

y=a∗x∗i

The increment (c) to reach the objective function (maximising the reduction in diarrhoea events) with a 3-dose schedule is a function of the change in the vaccine coverage rate (a_1_) and the extra vaccine efficacy (x_1_) obtained, compared with a 2-dose schedule, while the population incidence rate remains unchanged:

$$y+c=\left[\left(a+a_{1}\right)*\left(x+x_{1}\right)\right]*i$$

The change in coverage rate (a_1_) was assumed dependent on the relative price difference per dose between a 2- and a 3-dose vaccine schedule, given a fixed budget for vaccination. There will automatically be a link with the reduction in vaccine coverage rate, if the price difference per dose and per vaccine scheme decreases, as an increase in the vaccine efficacy (x_1_) is then required for the 3-dose schedule to keep its advantage over the 2-dose schedule. Sensitivity analysis should demonstrate what price difference, what vaccine coverage rate, or what vaccine efficacy difference would be required to achieve a change in the preference between the two dosing schedules. In addition, the change in health outcomes will affect the overall management cost of the disease. Changes in vaccine coverage rate and/or vaccine efficacy would be expected to affect the cost drivers for overall disease management costs, such as hospitalisation rate. To address this, we add an evaluation of the budget change for overall disease management as a relative value to the fixed budget for vaccination as an additional output variable in the sensitivity analysis.

## Results

### Analysis with fixed data

Tables 1 and 2 provide an example to illustrate the model. Table 1 shows the input data and Table 2 the modelled outputs. This hypothetical example assumed an annual birth cohort of 10,000 children who could be vaccinated. Based on the assessment of disease burden and the financial priorities, the health ministry is assumed to have allocated an annual budget of $200,000 for rotavirus vaccination. The annual incidence rate of rotavirus diarrhoea without vaccination was set at 0.3 per child per year for the at-risk period (from birth up to age 5 years) of the birth cohort, thus an average of 3,000 children per year would be expected to develop diarrhoea without vaccination.

**Table 1:** Input variables

**Parameter**	**Value**
Total vaccination budget	$200,000
Cost/dose for 2-dose vaccine schedule (strategy A)	$13.00
Cost/dose for 3-dose vaccine schedule (strategy B)	$10.00
Diarrhoea incidence rate per child per year	0.30
Vaccine efficacy for 2-dose vaccine schedule (strategy A)	0.60
Vaccine efficacy for 3-dose vaccine schedule (strategy B)	0.65
Number of vaccine doses for strategy A	2
Number of vaccine doses for strategy B	3
Population	10,000
Target vaccine coverage rate	75%
Average treatment cost per diarrhoea event	$50.00

**Table 2:** Modelled outputs

**Output variable**	**2- dose schedule (strategy A)**		**3- dose schedule (strategy B)**	
	**Value**	**%**	**Value**	**%**
Number of diarrhoea events expected with no vaccination	3000		3000	
Vaccine cost	$195,000		$225,000	
Vaccine cost as % of available vaccination budget		97.5%		112.5%
Budget shortfall	0		$25,000	
Number of people in target population not covered because of insufficient budget	0		833	
Vaccine efficacy difference		5%		
Vaccine cost difference				23.1%
Number of people covered by current budget	7500		6667	
Number of diarrhoea events in vaccinated children	900		700	
Number of diarrhoea events in non-vaccinated children	750		1000	
Total number of diarrhoea events with vaccination	1650		1700	
Treatment cost	$82,500		$85,000	
Gain (reduction in diarrhoea events compared with no vaccination)	1350	45.0%	1300	43.3%
Total cost	$277,500		$285,000	

Using the input variables shown in Table 1.

Under strategy A (n_a_ = 2) the cost per dose was set to $13, and thus the cost per course of vaccination was $26 (=$13*2). With a target coverage rate of 75%, the cost of vaccination was estimated at $195,000 (=10,000*0.75*$26) which represents 97.5% of the total available vaccination budget. As such, there would be sufficient budget (i.e. the budget would not be exceeded) and the target coverage rate would be reached. Assuming a vaccine efficacy of 60%, 900 of the 7,500 children in the vaccinated part of the cohort would be expected to develop diarrhoea, in addition to 750 of the 2,500 children in the unvaccinated part of the cohort, yielding a total of 1,650 diarrhoea cases. Thus, the health benefit gained by vaccination with a 2-dose schedule would be a reduction of 1,350 diarrhoea cases (a reduction of 45%) for the full birth cohort, compared with no vaccination (Table 2).

Under strategy B (n_b_ = 3), the cost per dose was set to $10 and thus the cost per course of vaccination was $30 (=$10*3). With the same target coverage rate of 75%, the total cost of vaccination would be $225,000 (=10,000*0.75*$30), representing 112.5% of the vaccination budget. Thus, there would be insufficient budget (a shortfall of $25,000). This shortfall implies that the budget would have run out with 833 children among the targeted population still to be vaccinated ($25,000/$30 = 833), and thus the available budget would be sufficient to vaccinate 6,667 children. Assuming a vaccine efficacy for the 3-dose schedule of 65%, 700 of the 6,667 children in the vaccinated part of the cohort would still develop diarrhoea, in addition to 1,000 of the 3,333 children in the unvaccinated part of the cohort, yielding a total of 1,700 diarrhoea cases. Thus, the health benefit gained by vaccination using a 3-dose schedule would be a reduction of 1,300 diarrhoea cases (a reduction of 43.3%), compared with no vaccination (Table 2).

Comparing the two strategies, although the cost per dose was 23% lower with a 3-dose schedule (strategy B), the cost per course was higher ($30 vs $26). The 2-dose schedule (strategy A) was not only cheaper overall ($195,000 vs. $200,000), but also resulted in a greater reduction in diarrhoea events (45% vs 43.3%), despite having a lower vaccine efficacy than the 3-dose schedule. This is because the lower cost per course with the 2-dose schedule would allow more children to be vaccinated within the available allocated budget.

### Sensitivity analysis

**Figure 1:**
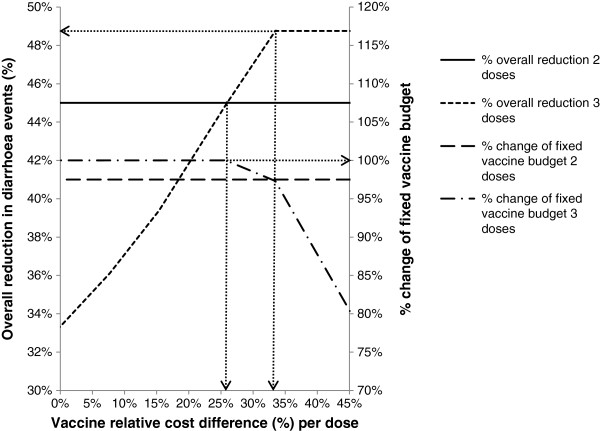
**Effect of the difference in vaccine cost per dose on budget and effect.** Reduction in diarrhoea events (left Y-axis) and relative change of vaccine budget constraint (=100%) (right Y-axis) as a function of the relative cost difference per vaccine dose. Assumptions: vaccine budget, $200,000; 2-dose vaccine efficacy (strategy A), 60%; 3- dose vaccine efficacy (strategy B), 65%; target vaccine coverage, 75%; 2-dose vaccine cost per dose (strategy A), $13.00; 3- dose vaccine cost per dose (strategy B), allowed to vary.

**Figure 2:**
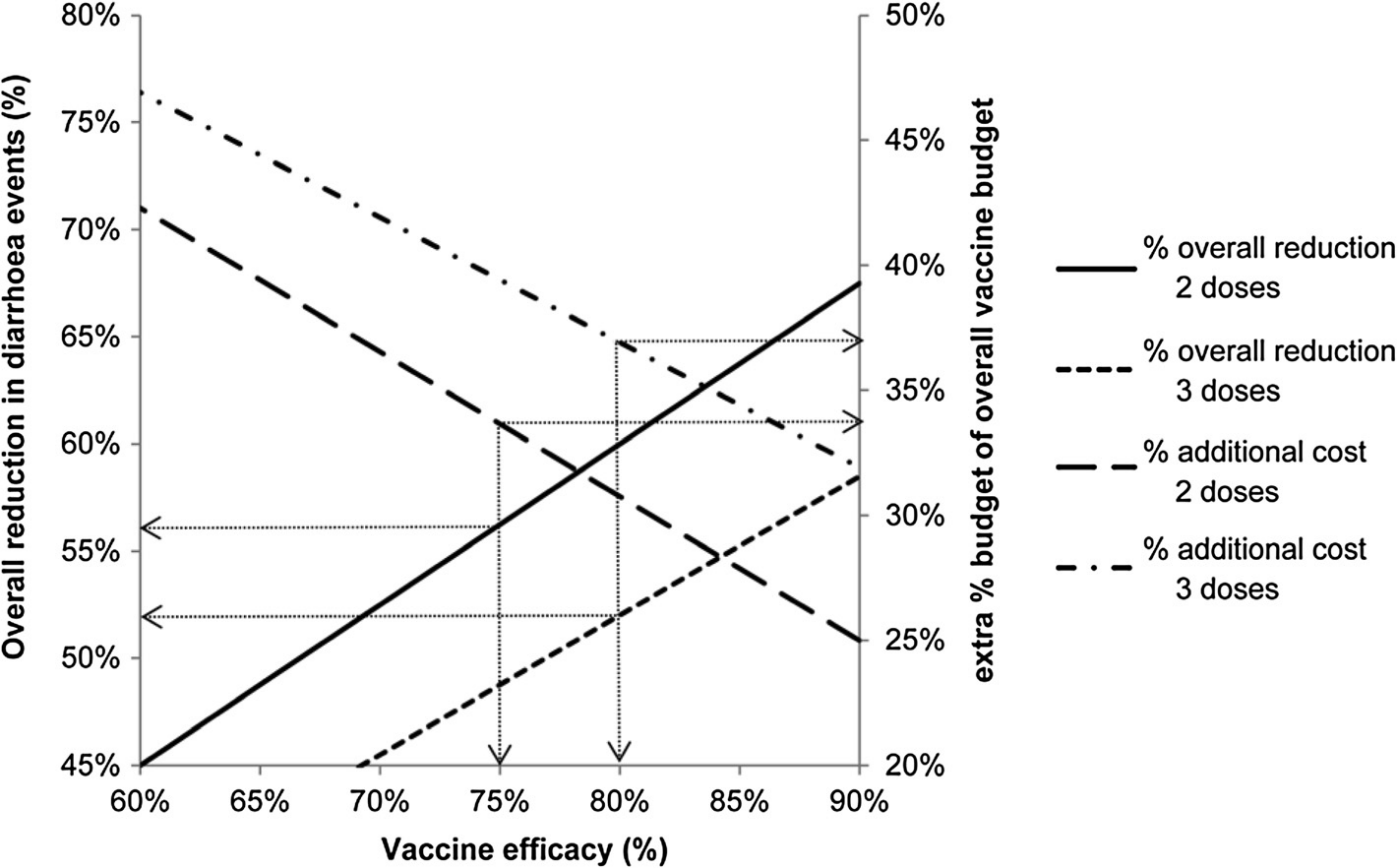
**Effect of vaccine efficacy on total cost and effect.** Total cost (right Y-axis) and effect (reduction in diarrhoea events, left Y-axis) as a function of vaccine efficacy. Assumptions: vaccine budget, $195,000; 2-dose vaccine efficacy (strategy A), 75%; 3-dose vaccine efficacy (strategy B), allowed to vary; target vaccine coverage, 75%; 2-dose vaccine cost per dose (strategy A), $13.00; 3-dose vaccine cost per dose (strategy B), $10.00.

**Figure 3:**
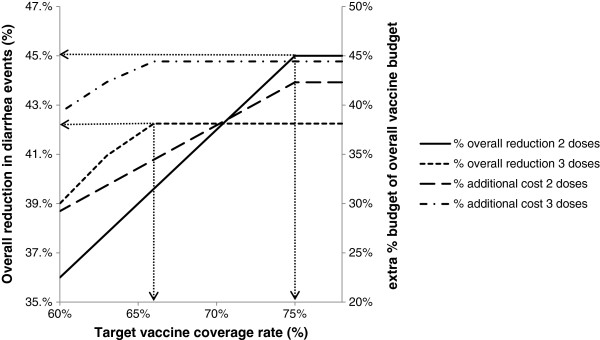
**Effect of target vaccine coverage rate on total cost and effect.** Total cost (right Y-axis) and effect (reduction in diarrhoea events, left Y-axis) as a function of target vaccine coverage rate. Assumptions: vaccine budget, $200,000; 2-dose vaccine efficacy (strategy A), 60%; 3-dose vaccine efficacy (strategy B), 65%; 2-dose vaccine cost per dose (strategy A), $13.00; 3-dose vaccine cost per dose (strategy B), $10.00; target vaccine coverage, allowed to vary.

Figures 1, 2, and 3 show the results of sensitivity analyses for a wide range of values tested.

Figure 1 presents the relationship between price per dose and the reduction in diarrhoea events and the total cost of vaccination under the assumptions listed in the legend. Consistent with the illustrative example shown in Table 2, with a 2-dose schedule a 45% reduction in diarrhoea events would be observed at a cost of 97.5% of the vaccination budget. Allowing the cost per dose for strategy B to vary, the figure illustrates that a large cost difference per dose of >33.3% would be required before the 3-dose schedule would become less costly; this occurs at the point at which the dash dotted line (- . –) crosses the dashed line (- -). A high cost difference per dose (>25.9% cheaper) would also be required for the 3-dose schedule to achieve a larger reduction in diarrhoea events than the 2-dose schedule; this occurs when the dotted line (…) crosses the solid line. Thus, a 2-dose vaccine schedule would always be the better choice when the relative cost difference does not exceed 25.9%.

There is a small area between a cost difference per dose of 25.9% and 33.3% where a larger reduction in diarrhoea events would be observed at a total vaccination cost within the fixed budget using the 3-dose strategy, while still remaining at the vaccine coverage rate of 75%. This area varies depending on other factors such as the vaccine efficacy difference. In situations where the 3-dose vaccine is cheaper, no additional reduction in diarrhoea events would be observed above the targeted vaccine coverage rate of 75%.

Figure 2 shows the effect of adding a treatment cost for a diarrhoea event, set at $50 per event, to estimate an overall cost for disease management (vaccination plus the cost of treating cases) with a vaccination cost of $195,000. For example, in Table 2, 1,650 cases of diarrhoea would be expected to occur with the 2-dose schedule, and the cost of treating these cases would be $82,500 (=1,650*$50). The total cost of the 2-dose strategy A would therefore be $277,500 (vaccine cost of $195,000 + cost of treating cases of $82,500). This value is 42.3% higher than the vaccination budget. Thus, under strategy A, assuming a vaccine efficacy of 60% a 45% reduction in diarrhoea events (solid line) could be achieved at a total disease management cost of 42.3% (dashed line) over the vaccination budget. Assuming a vaccine efficacy of 90%, a 67.5% reduction in diarrhoea events (solid line) could be achieved at a total disease management cost of 25% (dashed line) over the vaccination budget.

In Figure 2, it may appear counter-intuitive that a higher vaccine efficacy is needed with a 3-dose strategy versus a 2-dose vaccination strategy to obtain the same overall result. This reflects the higher vaccine coverage rate that can be achieved with a 2- dose vaccine strategy under budget constraint. In addition, the total budget would always be lower with a 2-dose schedule than with a 3-dose schedule when the difference in price per dose is lower than 33.3% (as shown in Figure 1 and discussed above).

Figure 3 demonstrates the effect of target coverage rate on the budget increment and the avoided diarrhoea events. On the left-hand side of the graph, where the target coverage rate is low, there would be sufficient budget for both strategies. Thus, the 3-dose strategy would prevent more cases (due to its higher efficacy) but at a higher cost. The cost for the 3- dose strategy (dash-dotted line) would exceed that for the 2-dose strategy (dashed line) at all coverage rates modelled. However, as coverage increases the number of events prevented by the 2-dose strategy (solid line) would overtake the number of events prevented by the 3-dose strategy (dotted line). This is because the maximum coverage rate achievable within the fixed budget would be higher for the 2-dose schedule (75%) than for the 3-dose schedule (66.7%). Thus, on the right-hand side of the graph where target coverage is high, the 2-dose schedule would prevent more cases than the 3-dose schedule at a lower cost.

## Discussion

The results of the modelling exercise presented here indicate that when the vaccination budget is constrained a 2-dose schedule for vaccination against rotavirus infection with the monovalent rotavirus vaccine would be expected to produce a larger reduction in disease events than a 3-dose schedule in most circumstances when using the same vaccine. This reflects the higher coverage rate that can be achieved with a 2-dose schedule than with a 3-dose schedule within a fixed budget. According to the model the 3-dose schedule would produce results superior to the 2-dose schedule only under the following conditions: large improvement in vaccine efficacy for the 3- dose schedule compared with the 2-dose schedule; large reduction in cost per dose for the 3-dose schedule compared with the 2-dose schedule; low target vaccine coverage rate. The effects of these parameters are closely intertwined. So a situation in which the 3-dose strategy would become superior to the 2-dose strategy may be achieved by a large change in one parameter alone, or by smaller changes in several parameters in combination. We will discuss each parameter separately.

A study in Africa reported vaccine efficacy against severe rotavirus gastroenteritis of 63.7% for a 3-dose schedule and 58.7% for a 2-dose schedule [21], a difference of 5 percentage points. However, it remains uncertain whether adding an extra dose truly improves the overall vaccine efficacy, as 3-dose vaccine efficacy studies using other vaccine products/candidates in low-resource environments have also reported lower efficacy estimates than in wealthier settings [13,14]. The difference in vaccine efficacy in our model was similar to the difference observed in the African study (65% versus 60%). Our results indicated that this magnitude of improvement in efficacy with the 3-dose strategy would result in an overall health gain (fewer diarrhoea events) compared with the 2-dose schedule only with a cost per dose for the 3-dose schedule of at least 25.9% lower than the cost per dose for the 2-dose schedule. The exact value will vary depending on the absolute vaccine efficacy values used, the budget available and the vaccine coverage rate, and thus will vary according to local circumstances. The smaller the gain in vaccine efficacy, the larger the cost difference per dose required.

Vaccine prices are often negotiated according to the total number of doses ordered by a country. An order for 60,000 doses intended to implement a 3-dose strategy covering 20,000 people may vary relatively little in price compared with an order also for 60,000 doses intended to implement a 2-dose strategy covering 30,000 people. Use of a budget optimisation tool may help decision-makers to identify the optimal strategy in their local environment, taking into account any changes in price as well as the expected change in vaccine efficacy and coverage.

The results presented here suggest that a 2-dose schedule is likely to be the optimal strategy, due to a higher vaccine coverage rate that the given budget allows. However, the vaccination budget is not the only factor influencing coverage rates. Other factors may include education, religious beliefs, attitudes to complementary and alternative medicine, gender-based inequity, civil unrest, the percentage of the population living in urban versus rural areas, accessibility of vaccination and other healthcare programmes, and financial factors [22-25]. Such issues are not insurmountable and high vaccine coverage rates can be achieved in low-income countries, as illustrated by high 3-dose diphtheria-tetanus-pertussis coverage rates in Kenya, Bangladesh and Sri Lanka [26]. Other interventions beyond the vaccination programme may be needed to improve the coverage rate, such as health education, better transportation, reduction in communication barriers to vaccinations, outreach to religious leaders and financial incentives.

Many studies have investigated the problem of optimal vaccine dosing schedules. Some have addressed the question from the opposite direction, evaluating whether a smaller number of doses can achieve the same clinical outcomes. For example, a 2-dose-plus-booster schedule for pneumococcal vaccination is accepted as having similar efficacy to a 3-dose-plus-booster schedule [27]. In the present analysis, as the effectiveness of the 2-dose rotavirus vaccine appears to be reduced in low-income countries the relevant question is whether adding one dose could improve clinical results. The strength of the model presented here is that it explicitly recognises the reality of a fixed budget. Adding an extra dose requires increasing the number of doses per vaccinee. Under a fixed budget this either requires an equivalent reduction in price to cover the same number of people or a corresponding reduction in coverage, or a combination of the two. An optimisation model can explore the question of whether increasing vaccine efficacy by adding an extra dose, or increasing coverage by using a 2-dose schedule, would be the best strategy to maximise the population health gains. It can also quantify the extra budget that would be required to achieve a larger health gain, providing a transparent method of assessing the best strategy for managing disease burden.

The model presented here could be applied to any question about the optimal dose schedule for any vaccine. For instance, the potential switch from a 2-dose to a 1-dose schedule for hepatitis A in Latin America is an important decision that requires careful choice of the optimal administration schedule [28]. The modelling exercise outlined here could provide useful guidance on this question that may be helpful for decision-makers. Additional refinements may be needed, as the present analysis did not use a dynamic model and did not consider the potential effect of an additional vaccine dose on herd protection, or differential waning rates for a 1-dose versus a 2-dose vaccine.

The model is simple in its construction and therefore has some limitations. For example, it does not take account of herd effects which may be important when considering the impact of changes in coverage. The higher coverage achievable with a 2-dose schedule compared with a 3-dose schedule within a fixed budget may lead to greater herd protection and thus to a larger difference in health benefit than estimated in the present model. Furthermore, the model does not cover changes in the timing of doses, effects of disease spread before the final dose, or natural immunity. In the case of rotavirus infection, natural immunity that develops with repeated infections is a competitor to vaccine-induced immunity, leading to a progressive reduction in the scope for vaccination to provide protection over time [29]. The model also does not take account of factors such as logistics and access to healthcare facilities to administer the additional dose[16]. However, in case of working under a fixed budget and increasing the number of doses per person, extra administration cost could be limited as a same person who already received vaccine doses will get an additional one. Things could be dramatically different with the reduction of the number of doses per person. The extra administration cost could then be much higher than in the previous situation because one has to reach additional people (increase the coverage rate) with the extra doses available.

Finally, we opted for a limited perspective in the analysis, namely the health care payer. We thought that essentially these people are most interested in the results when operating under a fixed budget. The societal perspective would only indicate that if a lower vaccine coverage rate was achieved with a 3-dose program, the societal cost could increase.

The optimisation approach here presented is very different from cost-effectiveness analysis. Cost-effectiveness analysis estimates the incremental cost per unit of incremental benefit to calculate an ICER value, and compares it with a threshold value considered to represent acceptable cost-effectiveness. However, to be meaningful this threshold must be locally defined, taking account of local circumstances. If the threshold is uncertain, the estimated ICER for an intervention may be of limited value in making a decision. Even if the threshold value is accepted, the ICER may not take account of infrastructure expansions required to implement an intervention. For example, a vaccination programme requiring a large increase in cold-chain capacity could be challenging for low-income countries, which in turn could result in a delay to vaccine introduction with consequences for expected health outcomes. Furthermore, an intervention requiring a substantial increase in expenditure – as may be likely with mass population interventions such as vaccination – may exceed the budget available, in which case it may be impractical to implement no matter how favourable the ICER.

The biggest difference between a cost-effectiveness analysis and an optimisation modelling is that in the latter it can take into account the coverage rate as an important variable to reach a certain health goal. In a cost-effectiveness analysis with a static model the vaccine coverage rate may not influence the ICER per se. This is different for a budget impact analysis where the vaccine uptake expressed through the coverage rate will impact the budget cycles. However budget impact analysis only informs about the financial spread over time and is not particularly linked to the goal or objective to be achieved within a defined period as optimisation modelling is pursuing.

Optimisation modelling, as presented in the exercise here, is clearer and simpler to understand [19]. Instead of a threshold value, it identifies the strategy that offers the largest health gain (in the case of a preventive intervention, the lowest number of disease events) within a fixed budget. This more closely reflects the reality of healthcare decisions. The number of available healthcare interventions continually increases, yet national healthcare budgets are not unlimited. It can be applied to simple problems such as the comparison between a 2-dose and 3-dose schedule for rotavirus vaccination illustrated here, or more complex issues such as human papillomavirus vaccination [20]. We may even consider the assessment of different vaccines against different diseases in order to prioritize their indication within a clear budget and time frame such as a multi-year vaccine portfolio management program [30].

Further research will be valuable to refine the simple model described here to take account of more complex issues such as herd protection effects or multi-criteria decision analysis designs.

## Conclusions

Optimisation modelling indicates that within a fixed budget and for the monovalent rotavirus vaccine, a 2-dose vaccine schedule would be expected to provide better health outcomes in most circumstances than a 3-dose schedule. The model can be used to quantify the conditions of changing dose schedules that would be optimal for any vaccine. It is a more transparent and powerful technique than the more conventional cost-effectiveness analysis for evaluation of the economic questions faced by decision-makers, because it explicitly recognises the budget constraint that is a reality in most healthcare systems around the world.

## Endnote

^a^Rotarix is a registered trade mark of the GlaxoSmithKline group of companies.

## Abbreviation

ICER: Incremental cost-effectiveness ratio

## Competing interests

BAS and DC are employees of the GlaxoSmithKline group of companies and own stock and stock options in the GlaxoSmithKline group of companies. MJP received payment for consultancy, lectures, development of educational presentations and membership of advisory boards, and his institution received research grants and payment for consultancy, from various companies in the pharmaceutical industry, including those producing rotavirus vaccines; i.e. the GlaxoSmithKline group of companies and Sanofi-Pasteur MSD. None of these was directly related to the current study.

## Authors’ contributions

BAS, DC and MJP developed the study methods, determined the model settings and inputs, and contributed scientific advice. BAS and MJP developed the model, reviewed the literature and conducted the statistical and sensitivity analyses. All authors contributed to the drafting of the manuscript. All authors had full access to the data and gave final approval before submission.

## Supplementary Material

Additional file 1 **A copy of the model used for the analysis in this manuscript with the original input values, as a Microsoft *Excel *workbook.** Click here for file
